# Application of Drug Testing Platforms in Circulating Tumor Cells and Validation of a Patient-Derived Xenograft Mouse Model in Patient with Primary Intracranial Ependymomas with Extraneural Metastases

**DOI:** 10.3390/diagnostics13071232

**Published:** 2023-03-24

**Authors:** Muh-Lii Liang, Ting-Chi Yeh, Man-Hsu Huang, Pao-Shu Wu, Shih-Pei Wu, Chun-Chao Huang, Tsung-Yu Yen, Wei-Hsin Ting, Jen-Yin Hou, Jia-Yun Huang, Yi-Huei Ding, Jia-Huei Zheng, Hsi-Che Liu, Che-Sheng Ho, Shiu-Jau Chen, Tsung-Han Hsieh

**Affiliations:** 1 Department of Neurosurgery, MacKay Memorial Hospital, Taipei 104, Taiwan; liang4617@hotmail.com (M.-L.L.);; 2 Department of Medicine, MacKay Medical College, New Taipei City 252, Taiwan; 3 Division of Pediatric Hematology-Oncology, Department of Pediatrics, Mackay Children’s Hospital, Taipei 104, Taiwan; 4 Department of Pathology, Shuang-Ho Hospital, Taipei Medical University, New Taipei City 235, Taiwan; 5 Department of Pathology, MacKay Memorial Hospital, Taipei 104, Taiwan; 6 Mackay Junior College of Medicine, Nursing, and Management, Taipei 112, Taiwan; 7 CancerFree Biotech, Ltd., Taipei 114, Taiwan; 8 Department of Radiology, MacKay Memorial Hospital, Taipei 104, Taiwan; 9 Department of Radiation Oncology, MacKay Memorial Hospital, Taipei 104, Taiwan; 10 Hospice and Palliative Care Center, MacKay Memorial Hospital, Taipei 104, Taiwan; 11 Department of Pediatric Endocrinology, MacKay Children’s Hospital, Taipei 104, Taiwan; 12 Division of Pediatric Neurology, Department of Pediatrics, MacKay Children’s Hospital, Taipei 104, Taiwan; 13 Department of Medical Research, Mackay Memorial Hospital, Tamshui Branch, New Taipei City 251, Taiwan; 14 Joint Biobank, Office of Human Research, Taipei Medical University, Taipei 110, Taiwan

**Keywords:** ependymoma, extraneural metastasis, circulating tumor cells, patient-derived xenograft, drug screening

## Abstract

Primary intracranial ependymoma is a challenging tumor to treat despite the availability of multidisciplinary therapeutic modalities, including surgical resection, radiotherapy, and adjuvant chemotherapy. After the completion of initial treatment, when resistant tumor cells recur, salvage therapy needs to be carried out with a more precise strategy. Circulating tumor cells (CTCs) have specifically been detected and validated for patients with primary or recurrent diffused glioma. The CTC drug screening platform can be used to perform a mini-invasive liquid biopsy for potential drug selection. The validation of potential drugs in a patient-derived xenograft (PDX) mouse model based on the same patient can serve as a preclinical testing platform. Here, we present the application of a drug testing model in a six-year-old girl with primary ependymoma on the posterior fossa, type A (EPN-PFA). She suffered from tumor recurrence with intracranial and spinal seeding at 2 years after her first operation and extraneural metastases in the pleura, lung, mediastinum, and distant femoral bone at 4 years after initial treatment. The CTC screening platform results showed that everolimus and entrectinib could be used to decrease CTC viability. The therapeutic efficacy of these two therapeutic agents has also been validated in a PDX mouse model from the same patient, and the results showed that these two therapeutic agents significantly decreased tumor growth. After precise drug screening and the combination of focal radiation on the femoral bone with everolimus chemotherapy, the whole-body bone scan showed significant shrinkage of the metastatic tumor on the right femoral bone. This novel approach can combine liquid biopsy, CTC drug testing platforms, and PDX model validation to achieve precision medicine in rare and challenging tumors with extraneural metastases.

## 1. Introduction

Ependymomas are the third most common pediatric central nervous tumor, behind only astrocytomas and medulloblastomas [1]. The highest frequency of occurrence is in those younger than 5 years of age, and two-thirds of pediatric intracranial ependymomas are located in the posterior fossa [2,3]. It is one of the most challenging malignant brain tumors in children and has a 50–81% 5-year overall survival rate, despite the availability of combined maximal safe resection and adjuvant chemoradiation therapy [1,4,5]. The pathologies based on the latest WHO CNS5 classification list two molecularly defined types of supratentorial ependymoma, *ZFTA* and *YAP1* fusion; two molecularly defined types of posterior fossa ependymoma, PFA and PFB; and a spinal tumor with *MYCN* amplified and myxopapillary type [6]. Despite advances in multimodality treatments, around 50% of children experience recurrence or disease progression [7,8]. To treat the recurrence of residual tumors, reoperation and reirradiation therapy are the main therapeutic modalities that provide the benefit of local tumor control [9,10,11]. According to the consensus of the Children Oncology Group (COG) and the International Society of Pediatric Oncology (SIOP), the provision of focal radiotherapy (RT) to patients with an ependymoma can occur in children as young as 12 months of age, since there was no evidence of early cognitive impairment in a large St. Jude study [12]. However, limitations in terms of the maximum tolerable dose and the long-term complications of RT still limit its use in young children [9]. To assess the benefits of postirradiation chemotherapy, a randomized phase III trial (COG ACNS0831) was conducted in which patients were assigned to either maintenance chemotherapy with vincristine, cisplatin, cyclophosphamide, and etoposide or observation following RT. The early results of this randomized trial suggest that maintenance chemotherapy may be beneficial, with a 3-year event-free survival of 80% vs. 71% (1-sided *p* = 0.0121) observed in ependymoma patients who received postirradiaton chemotherapy (*n* = 114) vs. those who received RT only (*n* = 196), respectively [13,14]. From the short-term evidence available, the eradication of chemotherapy-resistant recurrent ependyma using current chemotherapies remains difficult [4,5]. 

Due to the distinct treatment strategies used to treat the unique nature of ependymomas, modern healthcare has accumulated decades of experience through multicenter collaborations and the continuous investment of resources into research on translational medicine in order to identify the optimal timing and method to deal with recurrent tumors, further improving the prognoses of patients. The occurrence of extraneural metastases associated with intracranial ependymomas is extremely rare. According to a systemic review, only 48 cases have ever been reported in the literature [15]. The locations of metastases mostly involve the cervical or hilar lymph nodes, the lungs/pleura, and/or the scalp. The treatment options are limited, and no current protocol is available for extraneural metastatic ependymoma. Similarly to the treatment of primary ependymomas, multidisciplinary management strategies, including resection, locoregional radiotherapy, and/or systemic chemotherapy, are intended to delay a disease relapse. The median overall survival rate in patients with extraneural metastases is merely 3 months (range 0.1–36) [15]. Due to the abovementioned challenges associated with rare brain tumors in children, the development of treatment guidelines based on clinical trials cannot be achieved in the short term. The selection of an appropriate drug therapy should be based on the molecular characteristics of recurrent tumors to formulate personalized precision medicine strategies.

In peripheral blood samples of patients who have developed metastases, liquid biopsies containing circulating tumor cells (CTCs) can be used to provide a real-time and minimally invasive source of tumor cells [16,17]. In recent years, the collection of CTCs has expanded to liquid biopsies of patients with head and neck cancers [16], breast cancer [18], non-small-cell lung cancer [19], and prostate cancer [20]. The correlation between the drug sensitivity profiles of circulating tumor cell-derived organoids and clinical treatment responses was validated recently in a patient with pancreatic duct adenocarcinoma [17]. The application of CTCs to brain tumors has seldom been evaluated, since extraneural metastases rarely occur. Such obstacles are overcome by the use of highly specific staining techniques targeted at GBM-derived CTCs. Through advancements in methodology, more than 85% of 42 primary diffuse glioma and 8 recurrent glioma patients were found to have detectable CTCs. The presence of CTCs was confirmed to be a common property in the course of glioma [21].

To the best of our knowledge, CTCs have not yet been reported in ependymomas. Here, we present the application of a CTC drug testing model in a six-year-old girl with primary ependymoma on the posterior fossa. She suffered from tumor recurrence with intracranial and spinal dissemination at 2 years after her first operation and extraneural metastases in the lungs, mediastinum, and distant femoral bone at 4 years after initial treatment. Since no drugs are currently available to treat this stage of disease, CTCs were cultured and screened for clinical use as off-label drugs as salvage therapy. The drug screening platform selected two potential agents, everolimus and entrectinib, which significantly decreased CTC viability after treatment. The efficacy of the selected agents was also validated in a patient-derived xenograft (PDX) mouse model based on the same patient and significantly decreased tumor growth was confirmed. After the individualized precise drug screening, a combination of focal radiation on the femoral bone and everolimus treatment was performed. The whole-body bone scan showed significant shrinkage of the metastatic tumor on the right femoral bone after 3 months of treatment.

## 2. Materials and Methods

### 2.1. Clinical Data

Following institutional ethical approval (IRB No.20MMHIS323e), 12 patients aged younger than 20 with pediatric brain tumors, including 7 cases of diffuse glioma (WHO grades 2–4), 2 cases of posterior fossa ependymoma, type A (EPN-PFA), 2 cases of mixed germ cell tumor, and one case of hemangiopericytoma, received multimodality treatment at MacKay Memorial Hospital between January 2021 and July 2022. Peripheral blood (20 mL) was taken on the date of operation for the extraneural metastasis and, on the same day, it was sent to CancerFree Biotech Ltd. for the sorting and drug screening of circulating tumor cells. This technique is a fully pro bono entrustment. The representative tumor samples were harvested during the operation and sent for implantation into the patient-derived xenograft (PDX) mouse model. One of the EPN-PFA cases included in the study with a successfully established PDX mouse model proceeded to the testing of selected drugs. After confirming the efficacy of the selected drugs in the PDX model and obtaining the consent of the family members for off-label use, the agent was added to the combination of clinical salvage treatment.

### 2.2. Circulating Tumor Cell Sorting and Drug Screening

Isolation of the circulating tumor cells was performed as described previously [22]. Briefly, peripheral blood (20 mL) samples were collected in ETDA tubes and added to a LeucoSep tube (Greiner Bio-One International, Kremsmünster, Austria) with Ficoll-Paque (Merck, Kenilworth, NJ, USA) and centrifuged to obtain the PBMC fraction. The pellet was resuspended in phosphate-buffered saline (PBS) containing 1% BSA and 2 mM EDTA and enriched the CTC with RosetteSep^®^ CTC Enrichment Cocktail kit (Stem cell technologies, Cambridge, MA, USA) in accordance with the manufacturer’s instructions. The diluted sample was added on top of the Ficoll-Paque, and then centrifugation was performed. The enriched cells were harvested and suspended in DMEM/F12 medium containing EGF, bFGF, and B27 supplement (Thermo Fisher Scientific, Inc., Waltham, MA, USA). Cells were seeded onto binary colloidal crystals (BCCs). The medium was replaced in each well every four days, and the CTC tumor spheroids were visualized by microscopy. For verification of the CTCs, they were fixed with paraformaldehyde and permeabilizated using 0.1% Triton X-100. The primary antibodies anti-GFAP and anti-PanCK were used to analyze the CTCs, and cell nuclei were counterstained by DAPI. Images were examined and scanned using fluorescence microscopy.

### 2.3. Viability Assays for CTC Treatment

The cell viability was measured with a CellTiter-Glo^®^ Luminescent Cell Viability Assay (Promega, Madison, WI, USA). This assay is based on the quantitation of the ATP released from live cells. The detection limitation of this assay is 10 cells. The viability of the cells was measured by adding 100 µL of luminescent substrate to each well, incubating for 10 min at room temperature, and transferring the mixture to a white opaque 96-well microplate. The luminescence was then measured using a GloMax^®^ navigator microplate luminometer (Promega, WI, USA). CTC spheroids were allowed to proliferate in homemade plates for 4 weeks, and then organoids were harvested and equally plated in 96-well plates for 16 h of incubation. The anticancer drugs used in the clinical treatment were added to each well containing CTC-derived organoids. All anticancer drugs were purchased from MedChenExpress (Monmouth Junction, NJ, USA). The concentration of each drug used in the sensitivity test was selected according to the maximum (or peak) serum concentration (Cmax) in the body after administration of the drug, which is a standard measurement in pharmacokinetics [23]. Then, the cells were exposed to treatment with drugs and incubated for six days. The relative cell viability was calculated as the percentage of cells treated with drugs compared to untreated control cells.

### 2.4. Establishment, Verification, and Drug Testing of the Patient-Derived Xenograft (PDX) Mouse Model

With the approval of the institutional animal care and use committee (IACUC) of the VGH-TPE (IACUC No.: IACUC 2017-077 (approval period: 2018/1/1-12/31), IACUC 2018-257 (approval period: 2019/8/1-2022/07/31)) and the MacKay Memorial Hospital (IACUC No.: MMH-A-S-109-26 (approval period: 2020/4/20-2022/7/31), MMH-A-S-110-07 (approval period: 2021/8/1-2024/7/31)), the PDX mouse model was established as described previously [5]. Briefly, ependymoma tissues collected during surgery were diced into 2 × 2 × 3 mm^3^ fragments and implanted into the bilateral subcutaneous flank area of 6–8-week-old male NOD-SCID mice (F1). The xenograft was harvested for serial implantation after reaching approximately 1.5 cm^3^ and was additionally passaged two times in NOD/SCID mice (F2 and F3) to expand enough xenografts. The xenografts (F1–F3) were verified through hematoxylin and eosin staining and immunohistochemical (IHC) staining, and confirmed by a pathologist. IHC staining was performed using an antimitochondrial antibody (113-1, Novusbio, Littleton, CO, USA) and anti-Ki-67 (8D5, Cell Signaling, Danvers, MA, USA), anti-beat-III-tubulin (GT886, Genetex, Irvine, CA, USA), anti-GFAP (GA5, Cell Signaling), and anti-H3K27me3 (RM175, RevMab Biosciences, South San Francisco, CA, USA) antibodies. For drug treatment, the fourth generation of mice was established and stratified into three groups that were randomly assigned to be treated with DMSO, entrectinib (dissolved in DMSO, 7.5 mg/kg/day), or everolimus (dissolved in DMSO, 5 mg/kg, once every two days). Each treatment was administered for 19 days. Tumor volumes and mouse weights were recorded every 2 days. The tumor volume was calculated as follows: tumor volume (mm^3^) = [(length × width^2^)/2]. For the detection of downstream signal transduction, IHC staining was performed using anti-phospho-p70 S6 Kinase (AP1059, ABclonal, Woburn, MA, USA) and anti-phospho-AKT-Ser473 (D9E, Cell Signaling) antibodies.

### 2.5. RNA Sequencing

For RNA sequencing, total RNA was extracted from tumor tissues and xenografts in accordance with the manufacturer’s instructions. The quality and quantity of the RNA were assessed using a bioanalyzer and Qubit. Total RNA was purified with the poly-A antibody, and then mRNA fragmentation was performed. Fragment mRNA was ligated to an adaptor and a specific index for further amplification and sequencing (Illumina^®^ TruSeq^TM^ stranded mRNA, San Diego, CA, USA). The reads of raw data were mapped to the Hg19 reference using STAR (v2.6.1). The gene expression levels were assessed using RSEM (v1.3.1) to calculate the count of each gene. The normalized count was identified using the R (v3.6.1) package, DESeq2 (v1.26.0), and the correlation heatmap was drawn with ggplot2.

## 3. Results

### 3.1. Clinical Courses and Timeline of Recurrence

**Figure 1:**
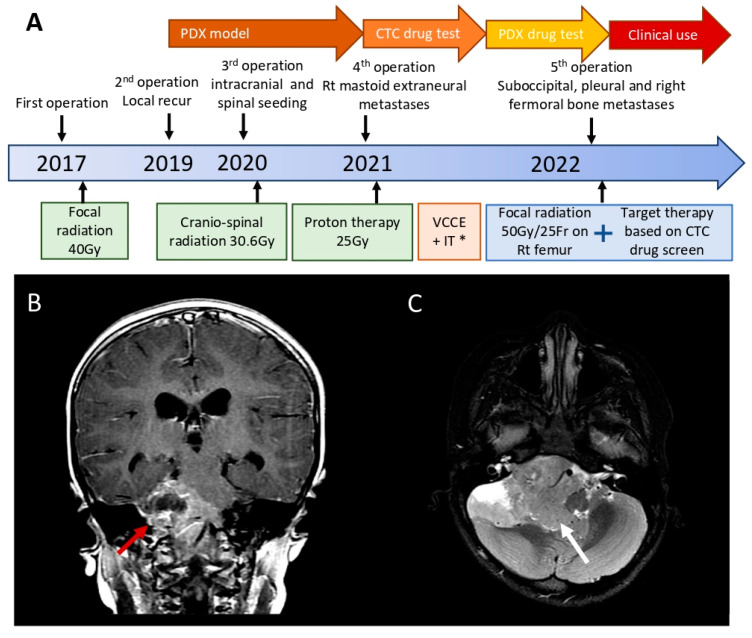
Primary ependymoma on the right cerebello-pontine angle with brain stem compression and extraneural metastasis in a 6-year-old girl. (**A**) Timeline of disease course and recurrence. * VCCE: vincristine, cisplatin, cyclophosphamide, etoposide; IT: intrathecal chemotherapy. (**B**) T1-weighted MR image with contrast enhancement showing the initial tumor mass wrapping the lower cranial nerve on the right cerebello-pontine angle (red arrow). (**C**) Axial view of the diffusion-weight image showing the iso-dense tumor mass compressing the brain stem (white arrow).

**Figure 2:**
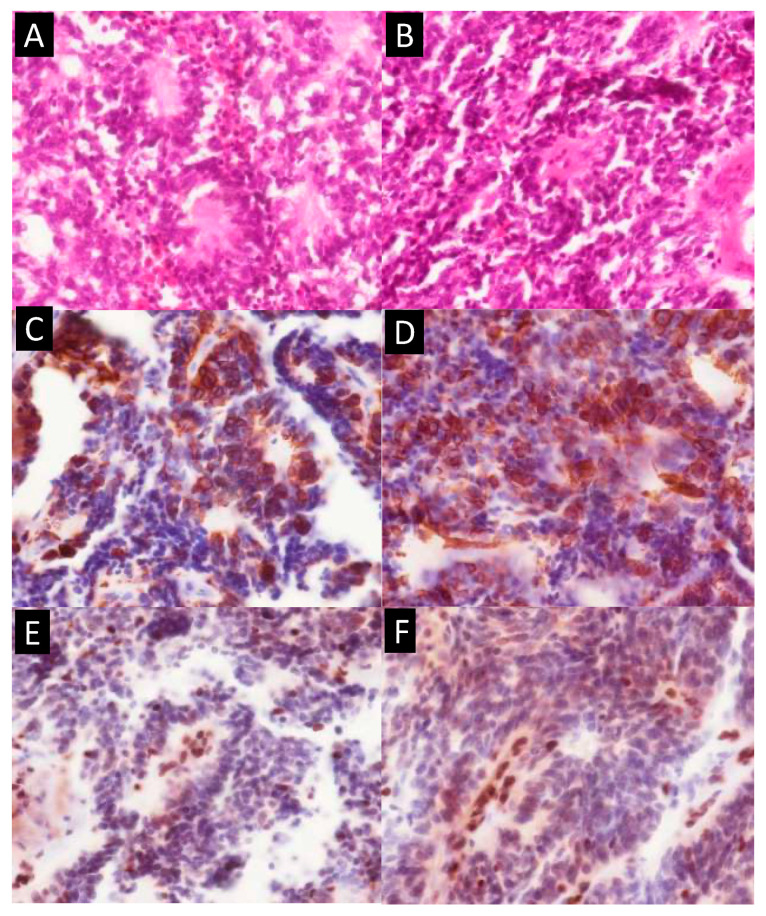
Primary and recurrent ependymomas of the posterior fossa, type A (EPN-PFA). (**A**,**B**) The neoplastic cells form perivascular pseudorosettes and ependymal rosettes in the primary (**A**) and recurrent (**B**) tumors (400× magnification). (**C**,**D**) Immunohistochemical stain for GFAP in the primary (**C**) and recurrent (**D**) tumors (400× magnification). (**E**,**F**) The tumor cells lost the expression of H3K27me3 (compared with the vascular endothelium cells as internal control) in the primary (**E**) and recurrent (**F**) tumors (400× magnification).

The patient’s clinical history and timeline of disease recurrence were reviewed (Figure 1A). The girl was brought to the emergency department due to a headache and vomiting when she was 3 years old. The first magnetic resonance image (MRI) of her brain showed a large heterogenous enhancing tumor mass located on the right cerebello-pontine angle with encasement of the brain stem, vertebra-basilar arteries, and lower right cranial nerves (Figure 1B,C). She received an emergent suboccipital craniotomy for resection of the tumor in December 2017, and the pathological diagnosis was anaplastic ependymoma (WHO grade III, based on 4th ed WHO Classification of CNS Tumor, 2016) (Figure 2A,C). This diagnosis was further classified as EPN-PFA with decreased expression of H3K27me3 (Figure 2E). Postoperative images showed near total resection of the tumor. The patient maintained a disease-free status for 2 years after adjuvant focal irradiation at 40 Gy, and then recurrence on the previous tumor bed on the right cerebello-pontine angle occurred in December 2019. The recurred tumor was reoperated on, and the tumor samples were implanted into a subcutaneous PDX mouse model (Figure 1A). A second recurrence on the left lateral ventricular and lumbar spinal dissemination was noted in June 2020 (Figure 2B,D,F). The patient underwent an aggressive surgical resection on the identifiable tumor locations, followed by cranio-spinal irradiation at 30.6 Gy/17fx and a local boost on the left lateral ventricle at 23.4 Gy/13 fx. Postirradiation adjuvant chemotherapy based on the COG ACNS0831 protocol with 1.05 mg vincristine (1.5 mg/m^2^), 10.5 mg cisplatin (20 mg/m^2^), 630 mg cyclophosphamide (1200 mg/m^2^), and 52.5 mg etoposide (100 mg/m^2^) at a dose of 75% were applied at intervals of 4–6 weeks for maintenance, and 10.5 mg intrathecal methotrexate (15 mg/m^2^), 21 mg hydrocortisone (30 mg/m^2^), and 42 mg cytarabine (60 mg/m^2^) were given monthly via lumbar puncture from June 2020 to July 2021. (Figure 1A).

**Figure 3:**
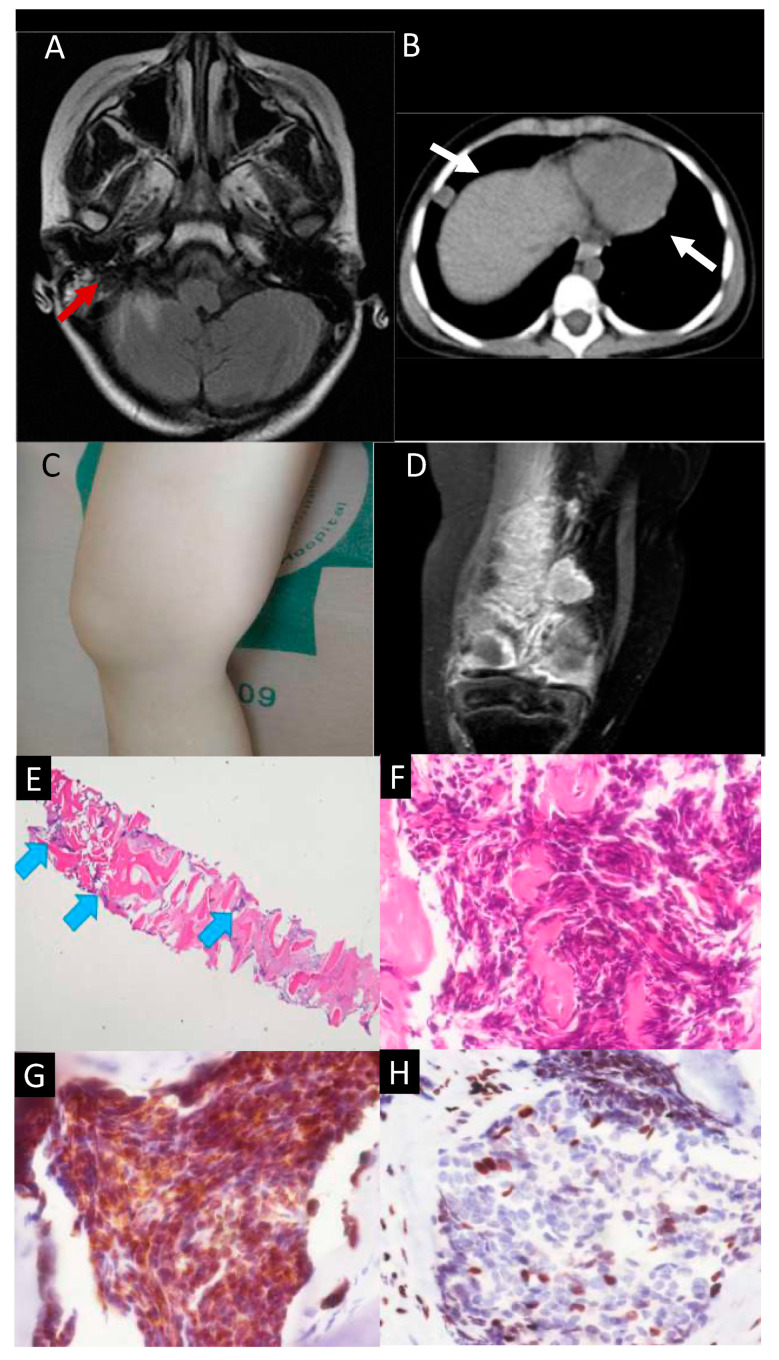
Primary ependymoma with extraneural metastases in (**A**) the mastoid sinus (red arrow) and (**B**) pleura and mediastinum (white arrow). (**C**) Gross appearance of the swelling thigh. (**D**) T1-weighted MR image with contrast of the right femoral bone showing an enhancing tumor mass on the femoral shaft and condyles. (**E**) Bone biopsy specimen showing the foci of metastatic EPN-PFA (blue arrows) (20× magnification). (**F**) The hyperchromatic tumor cells in the bone marrow forming a rosette-like structure on the H&E stain (400× magnification). (**G**) Immunohistochemical stain for GFAP in the neoplastic cells (400× magnification). (**H**) The tumor cells lost the expression of H3K27me3 (compared with other non-neoplastic bone marrow cells that were used as an internal control (400× magnification).

The first extraneural metastasis in the right mastoid sinus was noted on MR images taken in March 2021 (Figure 1A and Figure 3A) and confirmed by tumor resection after cortical mastoidectomy. The pathology revealed tumor cells with pleomorphic, hyperchromatic nuclei forming perivascular pseudorosettes resembling the morphology of ependymoma. Further immunohistochemical studies (GFAP and H3K27me3) confirmed the presence of the extraneural metastasis. Due to the unusual pattern of metastasis, peripheral blood samples were collected and sent for CTC sorting and drug screening. Proton therapy at 25 Gy in 5 fractions, followed by platinum-based systemic chemotherapy with carboplatin, etoposide, ifosphamide, and vincristine, was applied for salvage treatment [13]. Follow-up MR images taken after 3 months showed slight progression of the residual enhancing tumor on the right cerebello-pontine angle, so salvage gamma knife radiosurgery was performed in February 2022. However, the disease continued to progress, and swelling on the right thigh was observed in June 2022 (Figure 1A and Figure 3C). MR images of the right thigh showed a thickened cortical bone shaft with abnormal bone marrow enhancement, surrounded by linear subcutaneous stranding and perimuscular and intermuscular fascial thickening with fluid formation (Figure 3D). After tissue cone biopsy of the right femoral bone on July 2022 (Figure 3E), the pathology showed that neoplastic cells had infiltrated in the bone marrow. These cells were composed of hyperchromatic cells forming a rosette-like structure and were immunopositive for GFAP with reduced expression of H3K27me3, which is consistent with the occurrence of a metastatic EPN-PFA (WHO CNS grade 3, based on 5th ed WHO Classification of CNS Tumor, 2021) (Figure 3F,G,H). In the pleura and mediastinum, suspected metastatic nodules were identified in July 2022, and surgical resection with pathological confirmation was performed by a cerebrovascular surgeon in November 2022 (Figure 1A and Figure 3B). Despite multiple metastases and multiple operations, the neurological function of the patient remains close to normal, and she continues to be treated with the salvage drug. 

### 3.2. Confirmation of Circulating Tumor Cells in Ependymoma and Potential Efficacy of Entrectinib and Everolimus

**Figure 4:**
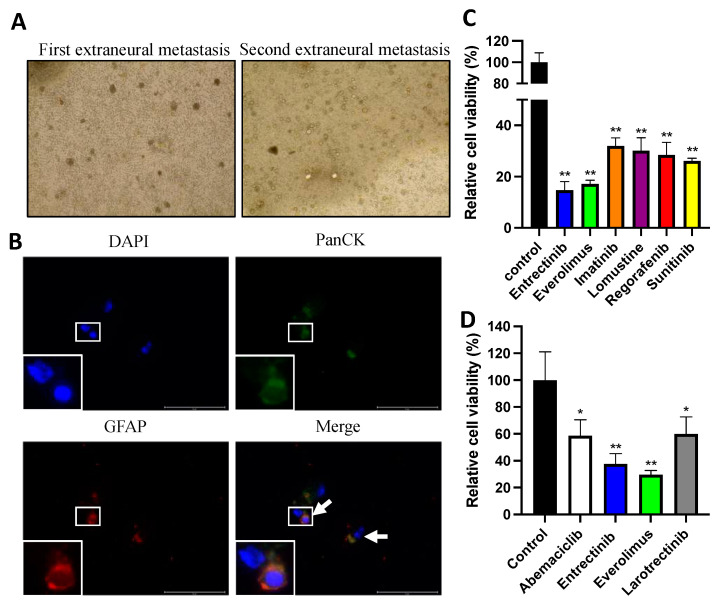
Drug screening of ex vivo circulating tumor cells derived from an ependymoma patient. (**A**) Representative bright-field images of organoids formed from the patient’s blood taken during the first (**left**) and second (**right**) operations of the extraneural metastases. (**B**) Immunofluorescence analysis confirming the circulating tumor cell expression of the markers of tumor cells (white arrows). PanCK: cancer-specific surface marker, GFAP: glial cell marker. Scale Bar: 75 μm. (**C**) Relative cell viability of CTC-derived organoids treated with different concentrations of chemotherapeutic agents from the peripheral blood taken during the first operation of the extraneural metastasis. Entrectinib: 3.13 μM, Everolimus: 0.064 μM, Imatinib: 7.5 μM, Lomustine: 4.3 μM, Regorafenib: 8.08 μM, Sunitinib: 0.181 μM. ** *p* < 0.01. (**D**) Relative cell viability of CTC-derived organoids treated with different concentrations of chemotherapeutic agents from the peripheral blood taken during the second operation of the extraneural metastasis. Abemaciclib: 0.588 μM, Entrectinib: 3.13 μM, Everolimus: 0.064 μM, Larotrectinib: 1.84 μM. * *p* < 0.05, ** *p* < 0.01.

Due to the observation of unusual extraneural metastases, peripheral blood was collected during these two operations for the sorting of circulating tumor cells (CTCs). The CTCs were enriched using a CTC enrichment kit and then cultured in the DMEM/F12 medium containing EGF, bFGF, and B27 supplements. CTC-derived spheroids were observed after 14 to 28 days (Figure 4A) and then confirmed by Pan-cytokeratin and GFAP immunofluorescence staining (Figure 4B). The CTC-derived spheroids were subsequently resuspended in a culture medium and seeded into 96-well plates to perform drug sensitivity assays. Due to the limited number of CTCs from the first operation on the extraneural metastases, we included targeted therapy drugs that are safe and clinically available for sick children in the drug screening test, including multi-kinase inhibitors (Regorafenib, Sunitinib), tyrosine kinase inhibitors (Imatinib), a mammalian target of rapamycin (mTOR) inhibitor (Everolimus), an alkylating agent (Lomustine), and a selective tyrosine kinase inhibitor (Entrectinib). After six days of treatment, we found that the CTCs were highly sensitive to everolimus and entrectinib (Figure 4C). A drug sensitivity assay of CTCs isolated from the second extraneural metastasis on the femoral bone was also performed using everolimus and entrectinib, as well as two other drugs, abemaciclib and larotrectinib. Abemaciclib is a CDK4/6 inhibitor that was shown to inhibit the growth of an ependymoma in our previous study [5]. Larotrectinib has similar targets to entrectinib, including TrkA, TrkB, and TrkC. Since the use of entrectinib is not approved for children by the Taiwan Food and Drug Administration (TFDA), we used larotrectinib and tested its cytotoxicity efficiency. The assay showed that the CTCs were more sensitive to everolimus and entrectinib than to abemacilcib and larotrecinib (Figure 4D).

### 3.3. Validation of the Efficacy of the Selected Drugs in the Patient-Derived Xenograft Ependymoma Model

**Figure 5:**
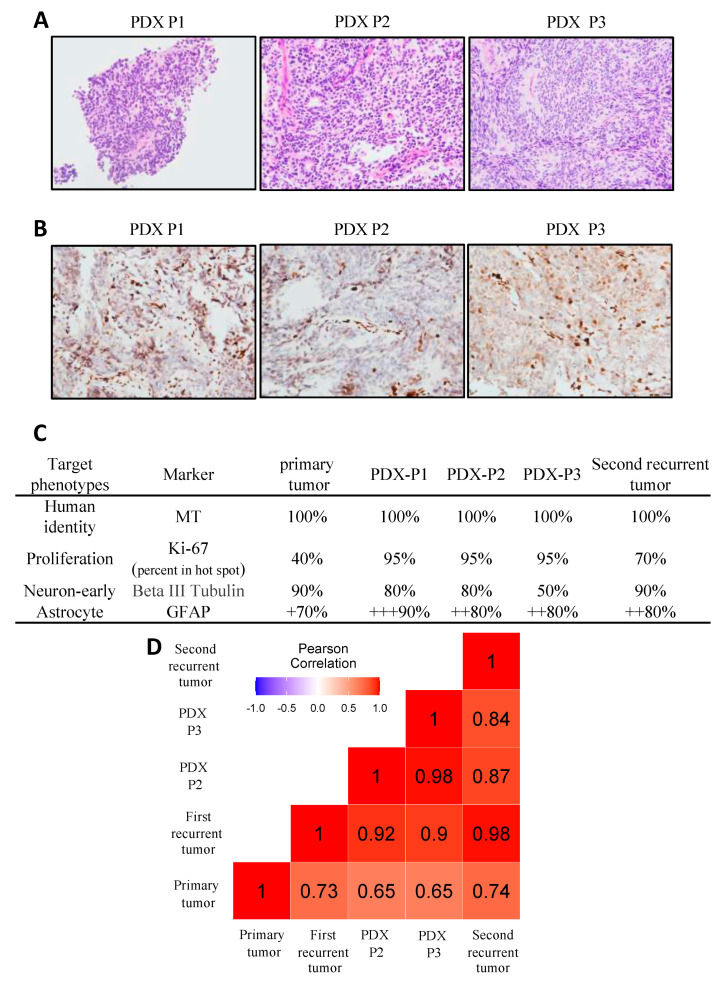
Patient-derived ependymoma xenograft validation. (**A**,**B**) IHC analyses confirmed the H&E (**A**) and H3K27me3 (**B**) results in all xenografts. PDX-P1: The first generation. PDX-P2: The second generation. PDX-P3: The third generation (400× magnification). (**C**) Summary of the IHC features of the patient’s tumors and all xenografts. +: weak staining, ++: moderate staining, +++: strong staining. (**D**) The matrix represents the relationships between the primary tumor, recurrent tumors and xenografts. A higher number indicates a closer relationship.

The PDX model was established from the tumor sample of the first recurrence in December 2019 (Figure 1A). We harvested the xenograft tumors from first-generation mice and reimplanted a piece of the tumor into a subcutaneous flank space of the recipient mouse to generate the next generation. The remaining tumors were used to determine whether the xenograft tumors replicated the histopathological and molecular phenotype of the first recurrent tumors by IHC staining and mRNA sequencing. The H&E stains of all xenografts displayed a perivascular pseudorosette and an ependymal rosette, suggesting that the pathologic diagnosis presented a similar morphology to the primary and recurrent tumors (Figure 5A). All xenograft samples also showed a remarkable reduction in histone 3 lysine 27 trimethylation (H3K27me3) expression in most tumor cell nuclei (compared with the vascular endothelium cells used as an internal control) (Figure 5B). Furthermore, the recurrent tumor and different generations of the xenograft samples displayed similar immunoprofile patterns to that of the primary tumor (Figure 5C). The mitochondria antigens (marker: MT) suggest that all xenograft tumors originated from human tumor tissues. The expression of the glial marker, GFAP, and the early neuronal differentiation marker, β III tubulin (marker: Tuj1), was also observed in all xenograft tumors. Interestingly, extremely high levels of the Ki-67 proliferation index, levels even higher than that of the recurrent tumor, were observed in the xenograft tumors. To determine whether the molecular expression patterns of the xenografts were similar to those of the patient’s tumors, we used Pearson correlation coefficients to calculate the similarity between the parental tumors and xenografts. The results showed that the first recurrent and second recurrent tumors had similar expression patterns (r = 0.98); however, all recurrent tumors showed lower levels of similarity in their expression patterns to primary tumors (r = 0.73–0.74), suggesting that clinical treatment may alter the molecular profiles of recurrent tumors. All xenograft generations exhibited higher correlations to the first- and second-recurrent tumors than to the primary tumor (r = 0.92–0.84) (Figure 5D). 

**Figure 6:**
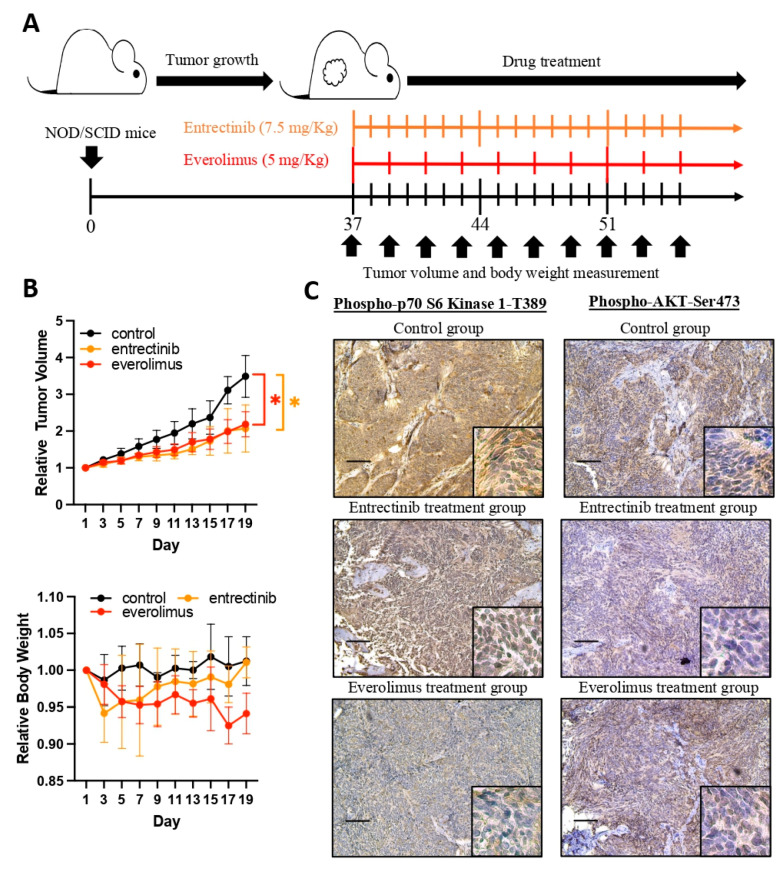
Entrectinib and everolimus treatments inhibit the growth of patient-derived ependymoma xenografts in vivo. (**A**) The drug treatment schedule: entrectinib (7.5 mg/kg, every day), everolimus (5 mg/kg, once every two days), or saline was injected (i.p.) into the patient-derived ependymoma xenograft for three weeks. The tumor volume and body weight were recorded every two days. (**B**) The figure shows the relative tumor volume and body weight compared with those at the first drug feeding. * *p* < 0.05. (**C**) IHC staining for the detection of phospho-p70 S6 kinase and phospho-AKT expression after treatment with entrectinib or everolimus. Scale bar: 100 μm.

To investigate whether entrectinib or everolimus can inhibit tumor growth, the fourth generation mice were established and treated with a vehicle, entrectinib (7.5 mg/kg, once daily) or everolimus (5 mg/kg, once every two days), and the tumor size and body weight were measured and recorded every two days (Figure 6A). As shown in Figure 6B, entrectinib and everolimus significantly inhibited tumor growth and no significant toxicity was observed. To elucidate whether entrectinib and everolimus treatment affected downstream signal transduction, we analyzed the phosphorylation status of the S6K and AKT pathways using IHC staining. Lower levels of S6K and AKT phosphorylation were observed after everolimus and entrectinib treatment, respectively (Figure 6C).

### 3.4. Everolimus Showed a Promising Effect When Given in Combined Salvage Therapy

**Figure 7:**
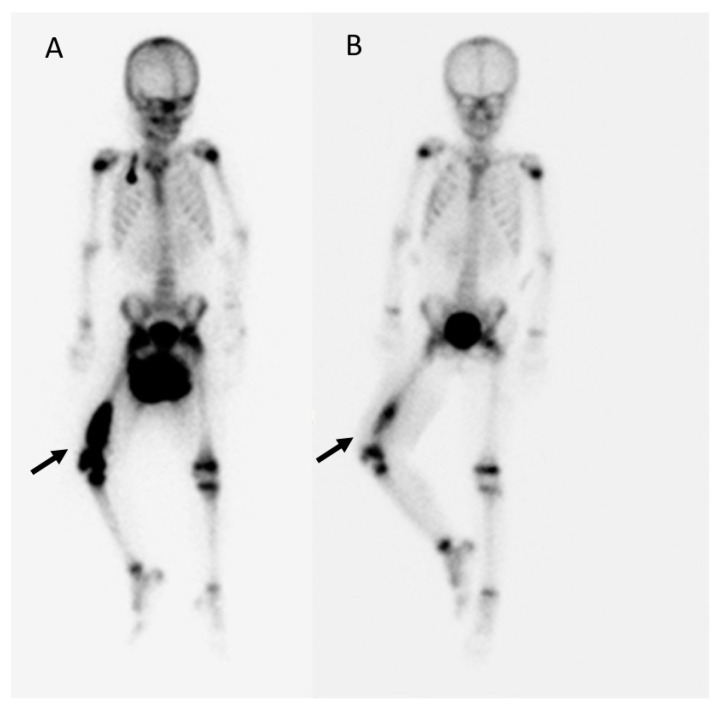
Tc-99m MDP whole-body bone scan showing significant regression of the extraneural metastasis on the right femoral bone after combined therapy (black arrow). (**A**) Before therapy on July 2022. (**B**) After combined therapy with 50 Gy focal irradiation and everolimus (2.5 mg per day) for 3 months (December 2022).

As no known drugs have been proven to be beneficial for the treatment of extraneural metastases of ependymoma, ex vivo drug screening of CTCs derived from an ependymoma patient was conducted. Two potential agents, everolimus and entrectinib, significantly decreased the CTC viability after treatment. The efficacy of the selected agents was validated in a PDX mouse model based on the same patient, and a significant decrease in tumor growth was confirmed. During this period, intensive modulated radiation therapy with a dose of 50 Gy/25 fx to the tumor bed and a boost of 16 Gy/8 fx to the gross lesion of the right distal femur was performed. Since the use of entrectinib had not yet been approved for children by TFDA, treatment with the combination containing the selected agent, everolimus (5 mg/day, 5 mg/tab, AFINITOR^®^, NORVATIS) began in September 2022, with the dose escalating to 5 mg per day after two weeks due to no significant side effects occurring. In December, the Tc-99m whole-body bone scan after the intravenous injection of 9.2 mCi MDP (methylene diphosphonate) showed significant regression of the extraneural metastasis on the right femoral bone after combined therapy (Figure 7A,B).

## 4. Discussion

Despite advances in multimodality therapies, intracranial ependymoma is still one of the most challenging malignant brain tumors in children. The recent advances in stereotactic radiosurgery and reirradiation strategies have improved the overall survival associated with these tumors [10]; however, around one third to half of all patients sustain a focal recurrence or distal seeding to the craniospinal axis, making the treatment of ependymoma more than just a one-time operation. The specific clinical factors that may contribute to recurrence include young age, the location of the tumor on the cerebello-pontine angle or the lateral medullary cranial nerve, brain stem compression, and early cranio-spinal seeding [4]. The posterior fossa group A (PFA) molecular subtype of H3K27me3 loss or EZHIP overexpression and a high Ki-67 index of more than 7% are also known to be associated with a poor prognosis [24]. After maximal safe resection, postoperative irradiation at a dose exceeding 45 Gy and the reirradiation of the relapsed tumor are the current mainstream treatment strategies [11,25]. The advantages of preirradiation chemotherapy were reported in patients with tumor resection of greater than 90% and a residual tumor of smaller than 1.5 cm^3^ in a COG study [26]; however, the clinical benefits for ependymoma of either regimen-based or high-dose chemotherapy treatment have not been proven yet. The usual course of PFA is similar to that used in our reported case. The 3-year-old girl initially suffered from an intracranial ependymoma located on the posterior fossa with wrapping around the brain stem and important neurovascular structures. We operated for more than ten hours in the first operation to achieve near-total resection of the tumor, and then achieved a two-year recurrence-free period after adjuvant focal radiation therapy. However, the first local recurrence and the second cranio-spinal dissemination occurred at two and a half years after the initial operation. There is an urgent need for the development of precision chemotherapy options, especially for patients with the abovementioned high-risk factors.

The extraneural metastasis of ependymoma was first reported by Maass L. et al. in 1954 [27], and many case reports continue to be published. According to a systemic review by Palmisciano P. et al. [15], the reported sites of metastasis include the scalp, cervical or hilar lymph nodes, kidneys, liver, lungs/pleura, long bones, vertebra, peritoneum, and other soft tissue. The common locations among the 48 patients included in this study were the cervical or hilar lymph nodes, the lungs/pleura, and the scalp, and only 7 cases of extraneural metastasis on the long bones have been reported. In addition to the above locations, our case also had metastases in the air cells in the mastoid antrum, which is an environment in which tumor cells do not easily grow. Such metastases demonstrate the recalcitrant growth properties of ependymoma cells, and new treatment modalities for these tumors still need to be developed. The treatment of femoral long bone metastases requires multiple considerations because the growing bone may be affected in children. Surgical resection of the femoral shaft can be performed, but it will inevitably lead to sequelae of leg length discrepancy. Therefore, it is urgent to preserve limbs with innovative therapeutic models. 

With the progress of molecular pathology, the latest classification based on the molecular subtype has become the standard and potential target of future treatment. New genetic testing or drug screening services are already available in many medical systems and markets, which positively impact clinical treatment availability for patients [28]. It is expected that, in the coming years, the expenditure on drugs in the field of oncology will exceed the cost of other medical types. A large number of innovative target therapies are being rapidly applied to clinical treatment, and the survival rate has been significantly improved for cancers such as breast cancer and non-small-cell lung cancer. However, high-priced innovative drugs must have a personalized screening platform to be cost-effective [29]. For the purpose of precision medicine in a challenging illness with multiple metastases, we combined a CTC drug screening platform and a personalized ependymoma PDX mice model in order to filter out the potentially dangerous drugs for this patient with extraneural metastases.

CTCs are cancer cells that have entered the blood circulation from the primary tumor. Clinically, the presence of CTCs is correlated with worse survival outcomes in many tumor types, including breast, colon, pancreas, prostate, and small-cell lung cancers [30]. The clinical application of circulating tumor cells has many purposes, including as therapeutic targets for metastatic tumors, early diagnosis, prognosis prediction, and real-time molecular phenotyping and minimal invasive liquid biopsies [31]. Furthermore, CTCs serve as a drug testing platform provided by living cells [32], and a correlation between CTC drug sensitivity profiles and the treatment response has been demonstrated in patients with pancreatic duct adenocarcinoma [17]. Recently, the application of CTCs to brain tumor treatment has been associated with great concern and has been evaluated. The main obstacle is the tiny amount of tumor cells that move through the blood–brain barrier, and this problem is overcome by the use of highly specific staining techniques targeted at GBM-derived CTCs. Due to advances in methodology, more than 85% of the 42 primary diffuse glioma and 8 recurrent glioma patients involved in our research had detectable CTCs [21]. Among the 12 pediatric brain tumor cases included in our study, the CTCs were sorted out and validated with positive GFAP immunofluorescence staining in 7 diffuse glioma and 2 ependymoma cases. Ependymomas from different locations have been shown to present differential molecular manifestations in many validated molecular analyses, and the immunohistochemistry of myxopapillary ependymomas has been reported to be focally positive for CK AE1/AE3, CAM5.2, and CK7 [33,34]. Furthermore, a comparative study of extra-axial and CNS ependymomas reported significant immunophenotypic differences with extra-axial cases preferentially expressing CK18 (100% vs. 20%), CK7 (80% vs. 10%), and CAM 5.2 (60% vs. 10%) [35]. Therefore, we sorted out the ependymoma-derived CTCs expressing both Pan-CK and GFAP (Figure 4B). Whether tumor cells with CK expression have a high potential to cause metastases has not yet been confirmed, and there may be differences in the morphology and immunohistochemistry evaluations of extraneural metastatic ependymomas, suggesting that these CTCs are derived from distinct precursors and/or are selected by the microenvironment. CTC-derived spheroids can then be used as a subsequent drug screening test. Only one child with an established ependymoma PDX model proceeded to PDX drug testing.

Two selected drugs, entrectinib and everolimus, showed higher levels of sensitivity than abemacilcib and larotrecinib in their ability to suppress the CTC-derived ependymoma spheroids. The efficacy levels of both agents showed significant tumor suppression in the personalized ependymoma PDX mouse model (Figure 6B). Everolimus, an inhibitor of mTOR, is a medication used as an immunosuppressant and as a target therapy for many different cancers. For subependymal giant cell astrocytoma associated with the tuberous sclerosis complex (TSC), everolimus is used as a multisystemic therapy that provides a reduction in tumor volume of more than 50% [36] and as an adjunctive therapy for TSC-associated partial seizures [37]. Many clinical trials are also underway to assess the combination of everolimus with other target therapeutic agents to treat malignant brain tumors in children [38,39]. The selected agents, entrectinib and Larotrectinib, have been approved by the United States FDA for the treatment of patients with NTRK gene fusion-positive advanced solid tumors, including young children with malignant gliomas [40]. Sustained responses of NTRK pathway inhibitors in cases of recurrent and disseminated ependymoma with the KANK1-NTRK2 fusion gene have also been reported [41]. To explore the potential actionable biomarkers, 13 known fusion genes, including NTRK1-3, ROS, and BRAF, were tested on the RNA-based next generation sequencing platform (ACT Fusion^TM^). The testing results were all negative in this patient, and the absence of NTRK fusion genes in clinical specimens is consistent with the inability of larotrectinib to effectively inhibit tumor cells due to its insensitivity (Figure 4D). As for the mechanism by which entrectinib inhibits the growth of ependymoma-derived CTCs and the PDX model (Figure 4C,D and Figure 6B), judging from the suppressed expression of phosphate AKT in PDX specimens after treatment (Figure 6C), it is likely to be achieved via non-NTRK pathways.

Thus far, the child has had a good response to their metastatic bone disease after a combination treatment with focal irradiation and everolimus (Figure 7). It is not possible to entirely attribute this treatment success to everolimus, as the radiation has certainly had some effect. Considering the indispensability of radiation therapy in the treatment of ependymoma, we adopted a simultaneous radiation and drug screening treatment to improve the timeliness of the treatment.

## 5. Conclusions

In order to carry out the real-time and nonsurgical exploration of preclinical drug testing for recurrent or metastatic malignant tumors, a drug screening platform that combined the use of circulating tumor cells and validation with a patient-derived xenograft model was employed. This precision medicine strategy can provide the optimal selection of effective drugs for the treatment of recurrent and resistant tumors.

## Data Availability

The data presented in this study are available on request from the corresponding author. The data are not publicly available due to the subject being a minor and a vulnerable group.
